# Mortality trends of breast and cervical cancer in Passo Fundo, Rio Grande do Sul: an analysis by age and schooling, 1999-2019

**DOI:** 10.1590/S2237-96222022000300021

**Published:** 2023-01-06

**Authors:** Vanessa Pecinato, Andréia Jacobo, Shana Ginar da Silva

**Affiliations:** 1 Universidade Federal da Fronteira Sul, Curso de Medicina, Passo Fundo, RS, Brazil; 2 Universidade Federal da Fronteira Sul, Programa de Pós-Graduação em Ciências Biomédicas, Passo Fundo, RS, Brazil

**Keywords:** Breast Neoplasms, Uterine Cervical Neoplasms, Time Series Studies, Mortality Records, Public Health

## Abstract

**Objective:**

to analyze the temporal trend of mortality due to malignant neoplasms of the breast and cervix from 1999 to 2019 in Passo Fundo, Rio Grande do Sul, Brazil.

**Methods:**

this was a time-series study based on data from the Mortality Information System; standardized rates were calculated according to age and schooling, and the temporal trend was assessed using Prais-Winsten regression.

**Results:**

the overall mortality coefficients for cervical cancer (β = -0.03; 95%CI -0.08;0.02) and for breast cancer (β = -0.006; 95%CI -0.02;0.01) were stable over the time series; in both types of neoplasms, a rising trend was identified in women with up to 7 years of schooling; on the other hand, a stationary trend was found in the majority of the age strata analyzed.

**Conclusion:**

older women and those with low levels of schooling had the worst prognosis.

**Study contributions**

**Main results**

Stability was found in the overall mortality coefficients for cervical and breast cancer over the course of the time series. However, different trend patterns were identified when the coefficients were assessed according to age and schooling.

**Implications for services**

Greater occurrence of mortality due to breast and cervical cancer in women with lower levels of schooling is a challenge for public health. We emphasize the need for policies that prioritize groups that are more vulnerable.

**Perspectives**

Monitoring breast and cervical cancer mortality trends is an important tool for the formulation of health promotion, prevention and follow-up measures for women at risk of a worse prognosis for the disease.

## INTRODUCTION

Incidence of malignant neoplasms of the breast and cervix is high and they are important causes of morbidity and mortality in the female population. According to data made available by the Global Cancer Observatory (GCO) of the World Health Organization (WHO), in 2020 there were more than 2 million new cases of breast cancer and more than 600,000 new cases of cervical cancer which, respectively, account for 24.5% and 6.5% of all new cancer cases in the female population worldwide. Regarding the total number of deaths from these two types of cancer, the GCO/WHO recorded the occurrence of 684,996 breast cancer cases and 341,831 cervical cancer cases, accounting for 15.5% and 7.7%, respectively, of the proportional distribution of all deaths from these two types of cancer in women.^1^

In Brazil, breast cancer incidence is the highest among all types of neoplasms in women in all the country’s macro-regions, with higher rates being found in the South and Southeast regions. The National Cancer Institute (Instituto Nacional de Câncer - INCA) forecast for 2022 is that there will be around 66,000 new breast cancer cases in Brazil, accounting for some 30% of all neoplasms in the female population - excluding non-melanoma skin cancer - which would correspond to an incidence rate of 43.7 new cases per 100,000 women.^2^ ^,^ ^3^ The breast cancer mortality rate was 14.2 deaths per 100,000 women in 2019, with higher coefficients in the Southeast and South regions.^4^

Cervical cancer is the third most common type of cancer in women. The forecast for 2021 was 6,710 new cases (7.5% of all neoplasms in women), with an estimated risk of 15.4 cases per 100,000 women.^2^ ^,^ ^5^ The Southern region, with an incidence rate of 12.6/100,000 women, ranks fourth in the analysis by region. The cervical cancer mortality rate was 5.3 deaths/100,000 women in 2019 for Brazil as a whole.^6^ Analysis done by region and state found that the state of Rio Grande do Sul had an estimated incidence rate of more than 4,000 new cases of malignant neoplasm of the breast, while incidence of malignant neoplasm of the uterine cervix was above 700 cases in a universe of 100,000 women.^3^ ^,^ ^5^

The implementation and subsequent expansion of cervical cancer screening activities in Brazil, which has enabled timely diagnosis and treatment, especially in the more developed regions of the country, has provided effective actions towards reducing incidence, increasing survival and reducing mortality from this type of cancer.^7^ A substantial part of cervical cancer incidence and mortality could be prevented by adopting a number of proven effective prevention measures, such as vaccination against human papillomavirus (HPV) in the 9 to 45 age group, access to health services, tobacco smoking control and use of early detection tests.^8^ However, middle- and low-income countries, such as Brazil, face challenges in implementing strategies that encourage and enable early diagnosis and treatment of cervical cancer and breast cancer, in order to reduce morbidity and mortality associated with these neoplasms.^7^

Due to the magnitude these neoplasms have reached and the consequent overburdening of the health system, the importance of intersectoral actions that facilitate access to health services and the improvement of the care offered to the affected population stands out.^9^ Therefore, research that analyzes neoplasm mortality over the long term, such as temporal trend studies of cervical and breast cancer mortality indicators, is opportune because it enables changes in the patterns of occurrence and time trends of these events to be explored,^10^ ^,^ ^11^ given that the study of mortality rates is a useful and efficient tool for understanding social determinants and evaluating the quality of health care and health service delivery.

The city of Passo Fundo is located in the Brazilian state of Rio Grande do Sul. It serves other municipalities in the region needing referrals to medium and high complexity health services. It is one of the largest health care provision cities in the state of Rio Grande do Sul, as well as being a referral center for the Brazilian National Health System (Sistema Único de Saúde - SUS). In this context, the present time series study will make it possible to quantify and compare health indicators related to cervical cancer and breast cancer, through a locoregional approach, involving different socio-demographic strata. As such, it will serve to inform the planning of public policies aimed at directing resources for prevention and treatment aimed at groups at greater risk.

The objective of this study was to analyze the temporal trend of mortality due to malignant neoplasms of the breast and cervix from 1999 to 2019 in Passo Fundo, Rio Grande do Sul, Brazil, according to age group and schooling.

## METHODS

This was a time series study, dedicated to analyzing the trend of mortality due to malignant neoplasms of the breast and cervix, taking the municipality of Passo Fundo as a unit of analysis. We included deaths from breast cancer and cervical cancer, by place of residence, reported on the Mortality Information System (*Sistema de Informações sobre Mortalidade* - SIM), of the Brazilian National Health System’s Department of Information Technology (*Departamento de Informática do Sistema Único de Saúde* - DATASUS), from 1999 to 2019, the underlying cause of which was coded as ICD-10 - C50 (breast cancer) and ICD-10 - C53 (cervical cancer), as per the 10th Revision of the International Statistical Classification of Diseases and Related Health Problems (ICD-10).

Passo Fundo is located in the north of the state of Rio Grande do Sul and has an estimated population of 203,275 inhabitants.^12^ It is considered to be the capital of the part of the state known as the Middle Plateau (*Planalto Médio*) and is one of the three main health service delivery cities in the Southern region of Brazil, providing referral services for over 66 municipalities in the north of Rio Grande do Sul, as well as cities in the west of the states of Santa Catarina and Paraná.

The data were retrieved from the information systems between April and September 2021. The variables studied were: year of occurrence of death (between 1999 and 2019), age group (in years: 20-29; 30-39; 40-49; 50-59; 60-69; 70-79; 80 or over) and schooling (in years of study: up to 7; 8 or more). “Age” and “schooling” variables with missing data were excluded from the tabulations and consequently, were also excluded from the respective calculations of the specific mortality rates in the strata of these variables.

The data on deaths due to malignant neoplasms of the breast and cervix were corrected according to the methodology proposed by the WHO,^13^ by adding to the crude reported deaths 50% of deaths the underlying cause of which was classified as “ill-defined” [symptoms, signs and abnormal findings of clinical and laboratory examinations, not elsewhere classified (R00-R99), except sudden infant death syndrome (R95)]. Deaths due to malignant neoplasms of the uterus were corrected by adding 50% the deaths classified as malignant neoplasm of uterus, part unspecified (CID-10 - C55).^14^

We also performed standardization of the breast and cervical cancer mortality rates, using the direct method, corrected by age group, using as a reference the population of women living in Passo Fundo, in each year analyzed, based on estimates obtained from the Brazilian Institute of Geography and Statistics (*Instituto Brasileiro de Geografia e Estatística* - IBGE).^12^ All rates were expressed in terms of the standard population size of 100,000 women.

The data exported from the SIM system and the population statistics were organized on electronic spreadsheets and then transferred to Stata Statistical Software, release 12.0, for data cleansing and analysis. The descriptive statistics were derived by calculating rates and proportions. We then took the number of deaths and resident population in the period in order to calculate the specific mortality rate due to malignant neoplasms of the breast and cervix, based on the following indicator: number of deaths due to the specific cause, in a given place and period, divided by the total population of the same place in the same period.

We used the Prais-Winsten generalized linear regression model to perform the temporal trend analysis. The models were built taking the dependent variable to be the mortality rates (β); and the independent variable to be the years of occurrence. The Durbin-Watson statistic was also applied in order to check for the presence of serial autocorrelation.

We then used the logs of the coefficients obtained through the regression analysis, and their respective 95% confidence intervals (95%CI), to calculate the annual percentage change (APC) for the total period, both overall and also for each age group and schooling group, as per the method proposed by Antunes & Cardoso.^15^ Finally, the mortality trend was interpreted as rising when the regression coefficients (β) and 95%CI showed positive values (p-value < 0.05); falling, when the coefficients and 95%CI showed a negative direction (p-value < 0.05); and stable, when no statistically significant difference was found between the β value and zero (p-value ≥ 0.05). All analyses were performed using Stata Statistical Software, release 12.0.

As this study used public domain free access secondary data, in which the participants were not identified, the study project was exempted from analysis by the Research Ethics Committees system, as authorized by the National Research Ethics Commission.

## RESULTS

**Figure 1:**
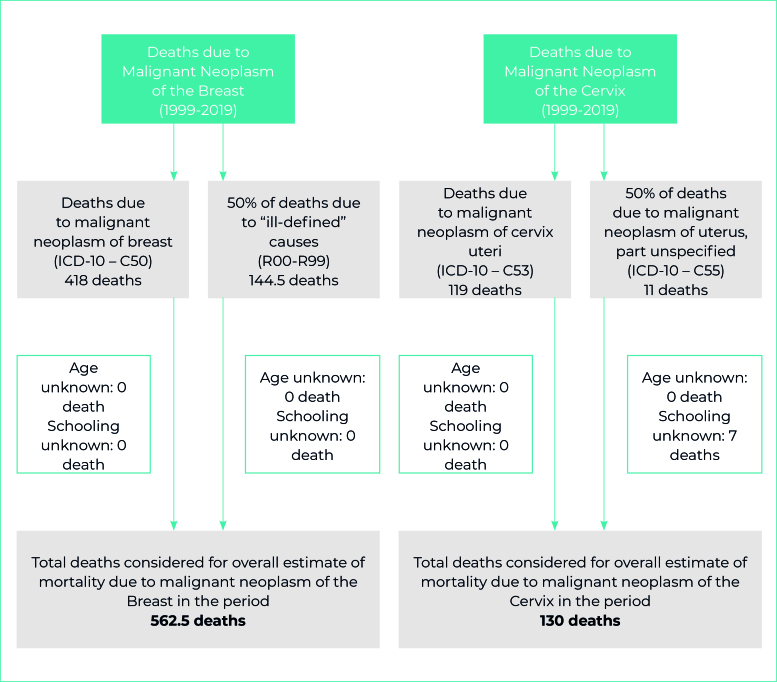
- Flowchart of the composition of deaths due to malignant neoplasm of the breast and cervical cancer, Passo Fundo, Rio Grande do Sul, 1999-2019

In the period analyzed, from 1999 to 2019, 119 deaths due to malignant neoplasm of the cervix uteri (ICD-10 - C53) and 418 deaths due to malignant neoplasm of the breast (ICD10 - C50) were found for women living in the municipality of Passo Fundo. The flowchart of the sample containing the description of the data, according to the parameters used to calculate the mortality rates, is detailed in Figure 1.

**Figure 2:**
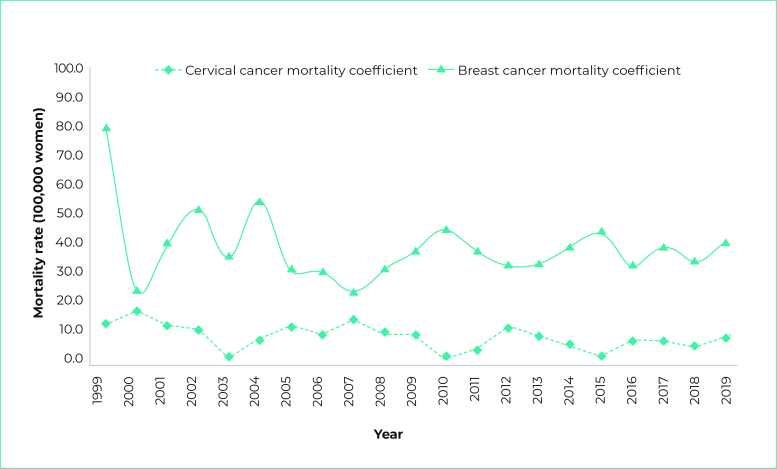
- Standardized mortality rates for breast cancer and cervical cancer, Passo Fundo, Rio Grande do Sul, 1999-2019

Over the time series, the overall mortality rates for cervical cancer were found to be stable (p-value = 0.153), with the highest rate in the year 2000, with 18.2 deaths per 100,000 women, and the lowest rate in 2003. Similarly, a stationary trend was found for breast cancer (p-value = 0.445). The highest breast cancer mortality rate was found in 2004, with 54.9 deaths per 100,000 women, while the lowest rate was in 2007 (24.0/100,000) (Figure 2).

a) Estimated based on Prais-Winsten regression.

The trend of the log-transformed cervical cancer mortality rates by age and schooling, as well as the annual percentage changes (APCs) and their respective 95%CIs, are shown in Table 1. Analysis of the rates by age group showed a falling mortality trend in the 50-59 age group (APC = -10.9; 95%CI -16.8;-6.7) and in the 60-69 age group (APC = -25.9; 95%CI -33.9;-16.8). No statistically significant changes were found for the remaining age groups over the time series, and a stationary trend was detected.

**Table 1:** - Cervical cancer mortality rate trend, by age group and schooling, Passo Fundo, Rio Grande do Sul, 1999-2019

Variables	Deaths (1999-2019)	Reference Population	Mean standardized mortality rate^a^ (1999-2019)	Coefficient β^b^ (95%CI^c^)	APC^d^ (95%CI^c^)	p-value^e^	Trend
Overall	130	97,713	9.3	-0.03 (-0.08;0.01)	-6.7 (-16.8;2.3)	0.153	Stationary
Age (in years)							
20-29	6	16,461	1.8	0.00 (-0.01;0.02)	0.9 (-2.3;4.7)	0.640	Stable
30-39	22.5	14,940	7.3	-0.02 (-0.07;0.04)	-4.5 (-14.9;9.6)	0.473	Stable
40-49	23	13,259	8.2	-0.01 (-0.05;0.04)	-1.4 (-10.9;9.6)	0.784	Stable
50-59	32	10,756	15.6	-0.05 (-0.08;-0.03)	-10.9 (-16.8;-6.7)	0.001	Falling
60-69	25.5	7,068	20.3	-0.13 (-0.18;-0.08)	-25.9 (-33.9;-16.8)	< 0.001	Falling
70-79	15.5	4,129	16.7	0.01 (-0.03;0.05)	2.1 (-6.7;12.2)	0.664	Stable
≥ 80	5.5	1,924	13.7	-0.00 (-0.06;0.06)	-0.7 (-12.9;14.8)	0.900	Stable
Schooling (in years of study)							
< 7	71	20,473	21.4	0.12 (0.07;0.16)	31.8 (17.5;44.5)	< 0.001	Rising
≥ 8	23	47,010	2.3	0.01 (-0.04;0.06)	1.9 (-8.8;14.8)	0.741	Stable

a) Number of deaths per 100,000 women; b) After logarithmic transformation; c) 95%CI: 95% confidence interval; d) APC: Annual Percentage Change; e) Prais-Winsten linear regression t-test, 5% significance level.

With regard to schooling, there was no statistically significant change in the cervical cancer mortality trend in women with more than 8 years of schooling (APC = 1.9; 95%CI -8.8;14.8), in the period, while in the case of women with up to 7 years of schooling, there was an increase in this trend, with an annual percentage increase of 31.8% (95%CI 17.5;44.5).

Table 2 shows the log-transformed breast cancer mortality rates and APCs, with their respective 95%CI, according to age group and level of schooling. The analysis of the rates by age group showed a falling mortality trend in the 40-49 age group (APC = -8.8; 95%CI -14.9;-2.3), in the 50-59 age group (APC = -4.5; 95%CI -8.8;-0.1), as well as in those aged 80 years or older, for whom an annual downward trend of 14.9% (95%CI -20.6;-6.7) was found. The trend was stationary for the remaining age groups.

**Table 2:** - Breast cancer mortality rate trend, by age group and schooling, Passo Fundo, Rio Grande do Sul, 1999-2019

Variables	Deaths (1999-2019)	Reference Population	Mean standardized mortality rate^a^ (1999-2019)	Coefficient β^b^ (95%CI^c^)	APC^d^ (95%CI^c^)	p-value^e^	Trend
Overall	562.5	97,713	39.5	-0.01 (-0.02;0.01)	-1.4 (-4.5;14.8)	0.445	Stable
Age (in years)							
20-29	5.5	16,461	1.6	-0.01 (-0.07;0.05)	-1.4 (-14.9;12.2)	0.682	Stable
30-39	17.5	14,940	5.9	-0.03 (-0.05;0.00)	-6.7 (-10.9;0.7)	0.074	Stable
40-49	65.5	13,259	23.9	-0.04 (-0.07;-0.01)	-8.8 (-14.9;-2.3)	0.021	Falling
50-59	136.5	10,756	62.6	-0.02 (-0.04;-0.00)	-4.50 (-8.8;-0.1)	0.049	Falling
60-69	101.5	7,068	20.3	-0.03 (-0.07;0.02)	-6.7 (-14.9;4.7)	0.225	Stable
70-79	96.5	4,129	113.3	0.01 (-0.03;0.04)	2.3 (-6.7;9.6)	0.607	Stable
≥ 80	138.5	1,924	404.5	-0.07 (-0.10;-0.03)	-14.9 (-20.6;-6.7)	< 0.001	Falling
Schooling (in years of study)							
< 7	280	20,473	90.9	0.14 (0.08;0.20)	38.0 (20.2;58.5)	< 0.001	Stable
≥ 8	133	47,010	12.7	0.11 (0.04;0.17)	28.8 (9.6;47.9)	0.003	Stable

a) Number of deaths per 100,000 women; b) After logarithmic transformation; c) 95%CI: 95% confidence interval; d) APC: Annual Percentage Change; e) Prais-Winsten linear regression t-test, 5% significance level

With regard to schooling, we found that regardless of the number of years of study, there was a rising trend in breast cancer deaths, with a greater increase among women with up to seven years of schooling (Table 2).

## DISCUSSION

This study found stability in the overall rates for cervical cancer and breast cancer in the city of Passo Fundo over the period analyzed. The analysis according to age groups and years of schooling showed that older women had the highest burden of mortality for these neoplasms, while those with low levels of education had the worst prognosis.

Overall mortality due to cervical cancer showed stability in the period from 1999 to 2019. However, when analyzing the rates according to age strata, a falling trend was seen in two of the seven age groups assessed. This result is similar to that found in an ecological study covering the period 2000-2016, in 456 primary healthcare center coverage areas in the municipality of São Paulo.^11^ Similarly, a national ecological study,^16^ covering the period 2003-2012, by age group and by macro-region, found a falling cervical cancer trend for Brazil as a whole, except for the Northern region.

Hypotheses exist as to the fall seen in recent years. The first may be related to increased access to screening, which enables diagnosis of precursor lesions and early treatment.^7^ The second hypothesis may reflect the strategies adopted in each healthcare territory, such as women actively seeking cytopathology examinations. It is known that adherence to this procedure is an important pillar of cervical cancer prevention.^17^ Both approaches can impact the reduction in morbidity and mortality due to this neoplasm. The decrease in mortality rates may also reflect the improvement in socioeconomic indicators found in recent years among the population, such as income and education.^18^

A study conducted in Aracaju which analyzed cervical lesion incidence and mortality between 1996 and 2015 ratifies this hypothesis by demonstrating a 3.8% reduction in each year in that period.^19^ Despite this progress, the scenario remains challenging for the control of this neoplasm, considering that in certain age groups the average crude mortality rate exceeds 20 deaths per 100,000 women. Moreover, a study published in 2020,^20^ about premature mortality attributed to cervical cancer in Brazil, revealed that 75% of deaths in women occurred between the ages of 30 and 69, signaling that prevention of this type of cancer has become a worldwide priority.

A similar result, regarding the stationary trend in the rates, was seen for breast cancer throughout the period we analyzed. One of the hypotheses for this stability may be the provision of less aggressive treatments, which provide greater safety and effectiveness regarding the course of the disease.^21^ Moreover, it is worth mentioning that favorable factors, such as screening and timely diagnosis, play an important role in this scenario, as well as the dynamics of health management during the entire process of screening, diagnosis, treatment and follow-up of women with malignant breast neoplasms.^21^ The municipality of Passo Fundo is a regional health referral center for the north of Rio Grande do Sul and municipalities in western Santa Catarina and Paraná. With regard to cancer care, Passo Fundo has a High Complexity Oncology Care Unit and a Cancer Institute, which has an innovative model of health management for oncology and hematology treatment, characterized by its pioneering, technological and multi-professional capacity, and is already consolidated within the SUS as an oncology referral service for the South of Brazil.

The implementation of these centers may have been reflected in the stability of the overall rates, considering that this is not the reality found in several other scenarios, which point to an increase in breast cancer mortality in recent years.^22^ ^,^ ^23^ This increase has been attributed to late diagnosis and gaps in access to treatment, especially in regions with low socioeconomic development ^22^ ^,^ ^23^

Different patterns were found when analyzing the rates according to age group. Breast cancer mortality showed a falling trend in the 40-49 and 50-59 age groups, and also in the group aged 80 or over. The results indicate that these differences may be directly influenced by sociodemographic and health characteristics, such as presence of comorbidities, socioeconomic status, availability and quality of health care. In addition, the stage of the disease at diagnosis acts as a predictor of prognosis and survival.^24^ ^,^ ^25^ There may also be association between increased coverage of mammography screening and increased breast cancer mortality, related to aspects such as overdiagnosis and overtreatment.^26^ ^,^ ^27^

This multifactorial etiology became evident when considerable inequities emerged when analyzing breast cancer mortality according to levels of education. Regardless of the number of years of schooling, there was a rising trend in deaths over the years. However, the number of deaths in women with up to 7 years of schooling was almost eight times higher when compared to deaths among those with more schooling, i.e. 8 years or more.

Women with low education and lower income are subject to limitations in access to health services and, consequently, their diagnosis is delayed, a fact that can result in greater exposure to death and increased risk of premature death.^28^ Confirming this hypothesis, a study conducted in the state of Sergipe with women undergoing chemotherapy treatment for breast cancer found that there were considerable disparities in access to health services.^24^ The delay in receiving test results, geographical barriers and difficulties in access to transport in order to have tests and treatment are factors that may explain these disparities in breast cancer mortality.

Also noteworthy is the trend found towards an increase in deaths due to malignant neoplasms in women with high levels of education. Possible hypotheses may be related to greater exposure to carcinogenic risk factors, due to their greater exposure to hormonal contraception, hormone replacement therapy during menopause and greater exposure to radiation, due to the greater number of mammograms performed, as well as lower protective factors, such as long menstrual history and nulliparity.^26^ ^,^ ^28^

The diversity and complexity of healthcare territories, social determinants, as well as the structure of the healthcare network, are important factors to be considered when analyzing morbidity and mortality indicators in the female population. The municipality of Passo Fundo, besides having 35 primary healthcare centers and referral hospitals,^29^ also has a Women’s Health Reference Center which opened in 2015. The various services available at the Center include multi-professional follow-up for women who have been discharged after cancer treatment, therapeutic groups before and after chemotherapy, as well as accompaniment of family members and guidance for families.^29^

This is one of the first studies to examine these estimates over a 21-year time span, in a municipality considered to be a reference in health care for the population of 66 municipalities in Rio Grande do Sul and several cities in western Santa Catarina and Paraná. Furthermore, it is worth noting that most of the studies published in the literature take into consideration national and/or regional analyses, a fact that can hide local-regional differences and limit the extrapolation of their results and conclusions to municipalities with different sizes and realities within the healthcare network.

Notwithstanding, this study has certain limitations that need to be considered. The use of secondary data is subject to variations in the completeness and quality of information. It is noteworthy that the low number of deaths from cervical cancer (ICD 10 - C53) in women living in Passo Fundo may be a reflection of underreporting and for this reason, may not match the reality of the disease, but rather reveal a failure in the definition of the cause of death, besides the problems identified in the quality of medical records.^8^

Another limiting factor is due to the multifactorial nature of malignant breast and cervical neoplasms, which have distinct characteristics. A higher proportion of more aggressive cancers may result in clusters of mortality, while access to better treatment consequently reflects in lower morbidity and mortality indicators.^30^ It is also noteworthy that mortality may be influenced by the geographic concentration of ethnic groups that are more likely to be genetically predisposed to cancer, as well as by missing information on the stage of diagnosis.^30^

Even with possible limitations, the study enabled us to find stability in the cervical cancer and breast cancer mortality rates in a municipality considered to be a regional health care provider in Southern Brazil. Important socio-demographic inequities were identified when stratifying mortality according to age and schooling.

Knowledge of temporal patterns enables elucidation of possible reasons for the behavior of these neoplasms, as well as enabling better planning and effective targeting of health promotion and prevention actions, since using mortality statistics makes it possible to know the health status of a population and the characteristics of the groups exposed to greater risk. We conclude that the evidence presented here can serve to inform policies for cancer prevention and oncology care in a municipality located outside Brazil’s major centers and characterized as a regional health care provider in Southern Brazil.

## References

[1] World Health Organization (2020). The Global Cancer Observatory. International Agency for Research on
Cancer [Internet].

[2] Instituto Nacional de Câncer José Alencar Gomes da Silva (2022). Estatística de câncer [Internet].

[3] Instituto Nacional de Câncer José Alencar Gomes da Silva (2022). Controle do câncer de mama: Incidência [Internet].

[4] Instituto Nacional de Câncer José Alencar Gomes da Silva (2022). Controle do câncer de mama: Mortalidade [Internet].

[5] Instituto Nacional de Câncer José Alencar Gomes da Silva (2022). Controle do câncer de colo do útero: Incidência [Internet].

[6] Instituto Nacional de Câncer José Alencar Gomes da Silva (2022). Controle do câncer de colo do útero: Mortalidade [Internet].

[7] Girianelli VR, Gamarra CJ, Silva GA (2014). Disparities in cervical and breast cancer mortality in
Brazil. Rev Saude Publica.

[8] Torre LA, Bray F, Siegel RL, Ferlay J, Lortet-Tieulent J, Jemal A (2015). Global cancer statistics, 2012. CA Cancer J Clin.

[9] Schlemmer JB, Castilhos LG, Lima SBS (2016). Políticas públicas e a atuação dos gestores frente ao câncer de
mama e do colo uterino- public politics and the actions of managers in front
to breast and uterine cervical cancer. Saúde (Santa Maria).

[10] Wang J, Lv H, Xue Z, Wang LU, Bai Z (2018). Temporal Trends of Common Female Malignances on Breast, Cervical,
and Ovarian Cancer Mortality in Japan, Republic of Korea, and Singapore:
application of the age-period-cohort model. Biomed Res Int.

[11] Bermudi PMM, PellinI ACG, Rebolledo EAS, Diniz CSG, Aguiar BS, Ribeiro AG (2020). Padrão espacial da mortalidade por câncer de mama e colo do útero
na cidade de São Paulo. Rev Saúde Pública.

[12] Instituto Brasileiro de Geografia e Estatística (2020). Cidades e Estados- Passo Fundo [Internet].

[13] Mathers CD, Bernard C, Iburg KM, Inoue M, Fat DM, Shibuya K (2003). Global Burden of Disease in 2002: data sources, methods and results.
Global Programme on Evidence for Health Policy Discussion Paper.

[14] Gamarra CJ, Valente JG, Silva GA (2010). Correction for reported cervical cancer mortality data in Brazil,
1996-2005. Rev Saude Publica.

[15] Antunes JLF, Cardoso MRA (2015). Uso da análise de séries temporais em estudos
epidemiológicos. Epidemiol Serv Saude.

[16] Vale DB, Sauvaget C, Muwonge R, Ferlay J, Zeferino LC, Murillo R (2016). Disparidades nas tendências temporais das taxas de mortalidade
por câncer do colo do útero no Brasil. Cancer Causes Control.

[17] Sousa SMS (2021). Saúde da mulher: busca ativa como ferramenta eficaz na cobertura
citopatológico. Revista Multidisciplinar em Saúde.

[18] Mullachery P, Macinko J, Silver D (2020). As reformas sanitárias no Brasil reduziram as desigualdades no
acesso a exames de câncer para mulheres?. J. Ambul Care Manage. Gestão de Cuidados.

[19] Lima MS, Brito EAC, Siqueira HFF, Santos MO, Silva AM, Nunes MAP (2020). Tendências do câncer do colo do útero e suas formas precursoras
para avaliar políticas de rastreamento em uma cidade de médio porte do
Nordeste brasileiro. PLoS One.

[20] Nascimento MI, Massahud FC, Barbosa NG, Lopes CD, Rodrigues VC (2020). Mortalidade prematura por câncer de colo uterino: estudo de
séries temporais interrompidas. Rev Saude Publica.

[21] Carioli G, Malvezzi M, Rodriguez T, Bertuccio P, Negri E, La Vecchia C (2017). (2017). Trends and predictions to 2020 in breast cancer mortality
in Europe. Breast.

[22] DeSantis CE, Bray F, Ferlay J, Lortet-Tieulent J, Anderson BO, Jemal A (2015). International Variation in Female Breast Cancer Incidence and
Mortality Rates. Cancer Epidemiol Biomarkers Prev.

[23] Li N, Deng Y, Zhou L, Tian T, Yang S, Wu Y (2019). Global burden of breast cancer and attributable risk factors in
195 countries and territories, from 1990 to 2017: results from the Global
Burden of Disease Study 2017. J Hematol Oncol.

[24] Gonçalves LLC, Travassos GL, Almeida AM, Guimarães AMDN, Gois CFL (2014). Barriers in health care to breast cancer: perception of
women. Rev Esc Enferm USP.

[25] Ramos JLS, Figueiredo FWS, Zuchelo LTS, Purcino FAC, Adami F, Gonçalves R (2020). Health Services, Socioeconomic Indicators, and Primary Care
Coverage in Mortality by Lower Genital Tract and Breast Neoplasias in
Brazilian Women during Reproductive and Non-Reproductive
Periods. Int J Environ Res Public Health.

[26] Diniz CSG, Pellini ACG, Ribeiro AG, Tedardi MV, Miranda MJ, Touso MM (2017). Breast cancer mortality and associated factors in São Paulo
State, Brazil: na ecological analysis. BMJ Open.

[27] Lôbo JLS, Silva MLC, Tomé TKBV, Souza CDF (2020). Mortalidade por câncer de mama feminino em Alagoas no período de
2001 a 2016: Análise de tendência e distribuição Espacial. Rev Bras Cancerol.

[28] Lundqvist A, Andersson E, Ahlberg I, Nilbert M, Gerdtham U (2016). Socioeconomic inequalities in breast cancer incidence and
mortality in Europe - a systematic review and meta-analysis. Eur J Public Health.

[29] Governo do Município (BR). Secretaria de Saúde (2017). Plano Municipal de Saúde 2014-2017 [Internet].

[30] Amin RW, Fritsch BA, Retzloff JE (2019). Spatial clusters of breast cancer mortality and incidence in the
contiguous USA: 2000-2014. J Gen Intern Med.

